# To Readers and Correspondents

**Published:** 1868-12-15

**Authors:** 


### *To Readers and Correspondents.*

The obituary notice of Dr. A. B. Shipman, late of Syracuse, N. Y., formerly Professor of Surgery in the Indiana Medical College, has not yet been received.——One of our subscribers, in order to complete his set of the JOURNAL, wishes to procure the volume for the year 1864. A liberal price will be paid for the same, on addressing the EDITOR.——The word LOOT seems to puzzle some of our readers. It is an Oriental word—much used during the Anglo-Chinese war, and the Sepoy rebellion, and literally signifies PLUNDER. Its applicability seems obvious.——The venerable Dr. Usher Parsons, of Providence, R. I., is dead. He was the last surviving medical officer who was present at Perry's victory. He had reached the ripe age of eighty.——Lallemand's *porte caustique* can be procured at the stores of our advertisers, Bliss & Sharp, or Chas. Degenhardt's.——We shall publish the matter, requested by our Quincy correspondent, next month.——The absence of our publisher, temporarily, from the city, must be our apology for delay in issue of the JOURNAL, and replies to letters. Every effort is being put forth to satisfy our friends, on all points.——The present number closes the volume, and a glance will show the large increase in its bulk over previous years. Examination of the *Index* will show a variety and importance in the matter contained, which should satisfy the demands of the most exacting subscriber. Arrangements for the coming year are such as to warrant the statement, that the volume for 1869 will surpass any of its predecessors.

